# The Role of Type 1 Conventional Dendritic Cells in Cancer Immunity

**DOI:** 10.1016/j.trecan.2018.09.001

**Published:** 2018-11

**Authors:** Jan P. Böttcher, Caetano Reis e Sousa

**Affiliations:** 1 Institute of Molecular Immunology and Experimental Oncology, Klinikum rechts der Isar, Technische Universität München, Ismaningerstraße 22, 81675 München, Germany; 2 Immunobiology Laboratory, The Francis Crick Institute, 1 Midland Road, London NW1 1AT, UK

**Keywords:** dendritic cells, cDC1, cancer immunity, tumor microenvironment, immune evasion, immunotherapy

## Abstract

Dendritic cells (DCs) are key orchestrators of immune responses. A specific DC subset, conventional type 1 DCs (cDC1s), has been recently associated with human cancer patient survival and, in preclinical models, is critical for the spontaneous rejection of immunogenic cancers and for the success of T cell–based immunotherapies. The unique role of cDC1 reflects the ability to initiate *de novo* T cell responses after migrating to tumor-draining lymph nodes, as well as to attract T cells, secrete cytokines, and present tumor antigens within the tumor microenvironment, enhancing local cytotoxic T cell function. Strategies aimed at increasing cDC1 abundance in tumors and enhancing their functionality provide attractive new avenues to boost anti-tumor immunity and overcome resistance to cancer immunotherapies.

## Highlights

Conventional type 1 dendritic cells (cDC1s) are a specialized subset of DCs with a unique role in cancer immune control.

Rejection of immunogenic cancers and the success of T cell–based immunotherapies in preclinical models requires functional cDC1s.

In human cancer patients, cDC1 abundance in tumor tissue is associated with cancer patient survival and the responsiveness to immune checkpoint blockade.

The access of cDC1s to tumor tissue is tightly regulated and involves chemokine secretion from intratumoral lymphocytes such as natural killer cells.

Tumor-derived factors impair anti-tumor immunity by limiting cDC1 accumulation, survival, and function within the tumor microenvironment.

## DC Biology in Cancer

Conventional DCs (cDCs) are especially adept at presenting exogenous and endogenous antigens to T cells and regulating T cell proliferation, survival, and effector function. This unique function of cDCs is crucial in the context of cancer, where cDCs take up antigens from tumor cells and present them to T cells within the tumor microenvironment (TME) or after migration to tumor-draining lymph nodes.

cDCs in mice and humans express CD11c and MHC class II and can be divided into two distinct subsets, cDC1 and cDC2 1, 2, although additional subsets can be delineated in both mice and humans 3, 4, 5. cDC1 depend on the transcription factors BATF3, IRF8, and ID2 for their development [5] and selectively express the chemokine receptor XCR1 and the C-type lectin receptor DNGR-1/CLEC9A 3, 6, 7, 8. Expression of the integrin αE (CD103) is also commonly used as an additional marker to identify cDC1 in mouse tumors, while BDCA3 is used for the same purpose in humans [9] (Box 1). cDC1sexcel at cross-presenting exogenous antigens (e.g., tumor antigens) to CD8^+^ T cells and are key cells for the generation of cytotoxic effector T cell responses. The importance of cDC1 in anti-tumor immunity is underscored by several studies with cDC1-deficient *Batf3*^–/–^ mice and other *in vivo* models of cDC1 depletion, which consistently display a loss of the ability to reject transplantable immunogenic tumors and are unable to support T cell–based immunotherapies such as adoptive T cell therapy or immune checkpoint blockade 10, 11, 12, 13, 14. In the above-mentioned models, loss of BATF3-dependent cDC1 cannot be compensated by other DC subsets or through BATF3-independent cDC1 development, for example, through cytokine-mediated induction of BATF and BATF2 [15]. However, cDC1s appear redundant for the success of poly(I:C) therapy and anthracycline chemotherapy in some mouse tumor models, arguing that other cells can compensate for lack of cDC1 in certain instances 16, 17.

Box 1 **Human cDC1**

In lymphoid and non-lymphoid organs, human cDC1s can be identified by BDCA3 expression and show a close relationship with mouse cDC1s at the gene expression level [9]. Similar to their murine counterparts, human cDC1s selectively express the C-type lectin receptor CLEC9A/DNGR-1 and XCR1, and this selective expression can be used in conjunction with BDCA3 expression to reliably identify these cells in human tissues. In addition to these phenotypic similarities, human and mouse cDC1s share many functional characteristics such as the efficient uptake and processing of dead cell–associated antigen for cross-presentation to CD8^+^ T cells and Toll-like receptor 3–induced production of IL-12 67, 68. However, IL-12 production is not as restricted to cDC1s in humans as in mice and can also be observed in cDC2s upon appropriate stimulation 69, 70.

Although human cDC1s only constitute a minority of myeloid cells in human tumors, similar to their murine counterparts, their presence in the TME is often associated with better survival of cancer patients 10, 26, 27. Furthermore, the abundance of cDC1s in human melanoma positively correlates with the responsiveness of these cancer patients to anti–PD-1 therapy [28]. These recent findings suggest an important role for cDC1 in anticancer immunity in humans.

Alt-text: Box 1

The development of cDC2 depends on the transcription factors RELB, IRF4, and ZEB2 2, 5, although additional subtypes of cDC2 have been characterized, including one that selectively depends on KLF4 [18]. cDC2s are commonly distinguished from cDC1s by their preferential expression of CD11b and CD172a. However, these markers do not suffice to reliably identify cDC2s in inflamed tissues or tumors as their expression is shared with other CD11c^+^MHCII^+^ myeloid cells such as macrophages and monocyte-derived DCs, which differ from cDCs 19, 20. Whereas cDC1 can be accurately identified by selective expression of molecules such as DNGR-1 or XCR1, proteins uniquely expressed by cDC2 have not yet been identified, hindering the development of models for selective detection and/or depletion of cDC2s in tumors. This might be one reason why knowledge about the behavior of cDC2s in tumors and their role in anti-tumor immunity is still limited. It is often assumed that cDC2s are predominantly involved in antigen presentation on MHC class II to CD4T cells in tumor-draining lymph nodes, similar to their role in microbial infection [2].

In this review article, we discuss the unique role of cDC1 in cancer immune control, focusing on the mechanisms and molecular pathways that enable cDC1 to accumulate in tumors, orchestrate anti-tumor immunity after migration to lymph nodes, and support immunity within tumor tissue. We further indicate how different aspects of cDC1 function are inhibited by immunosuppressive factors present within the TME. We refrain from discussing the pathways that lead to DC activation such as the recognition of damage-associated molecular patterns from dying tumor cells, which are important for ensuring DC functionality but have received ample coverage in the recent past 21, 22, 23.

## Access of DCs to Tumor Tissue

Compared to healthy tissue, cDC1s are under-represented in tumors [24] and constitute a small minority of intratumoral leukocytes in both mice and humans 10, 11, 25. Despite their scarcity, the overall tumor content of cDC1s, as assessed by cDC1-specific signatures in gene expression data and/or by flow cytometric analysis, positively correlates with cancer patient survival across multiple cancers and is predictive of the responsiveness to anti–PD-1 immunotherapy in melanoma patients 10, 26, 27, 28. Consequently, elevating cDC1 numbers in tumors by expansion with cytokines or through recruitment with chemokines (see below) leads to accelerated anti-tumor immunity, even in absence of added stimuli to promote cDC1 activation 11, 27.

The mechanisms that determine cDC1 abundance in tumors can involve chemokine-mediated recruitment, as well as chemokine-dependent retention and positioning of cDC1s within the TME. A major source of chemokines is the cancer cells themselves, because they secrete CXCL1, CCL2, and CCL20 to attract tumor-promoting immune cells such as monocytes, macrophages, regulatory T cells (T_reg_ cells), and T helper 22 cells 29, 30. Preferential production by tumor cells of chemokines that attract pro-tumorigenic immune cells might be one reason for the low cDC1 abundance observed in progressing tumors. Of note, absence of oncogenic signaling via the WNT/β-catenin pathway in the murine BRAF^V600E^/PTEN^–/–^ melanoma model allows production of CCL4 by tumor cells and causes increased accumulation of cDC1s within the TME [13].

**Figure 1:**
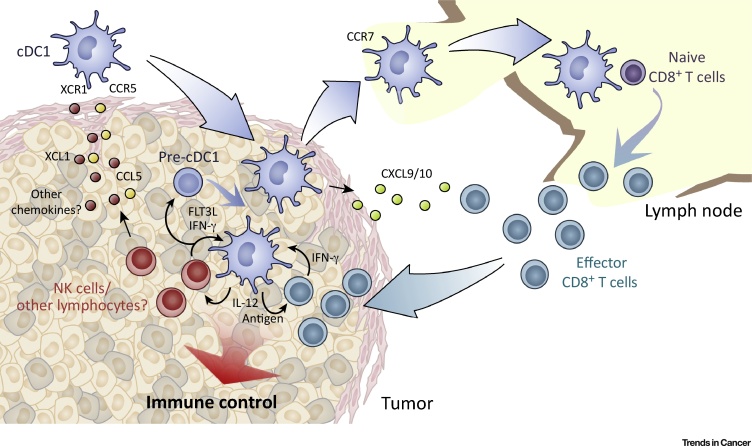
Orchestration of Cancer Immune Control by cDC1. Conventional type 1 dendritic cells (cDC1s) are recruited into the tumor microenvironment by chemokines such as XCL1 and CCL5, produced by intratumoral natural killer (NK) cells (and potentially other lymphocytes). NK cells further secrete the growth factor FLT3L, which supports the survival of cDC1s and might enhance local cDC1 differentiation from DC precursors. Within the tumor, cDC1s take up material from (dead?) tumor cells and are uniquely able to transport tumor antigens to tumor-draining lymph nodes for presentation to naive CD8^+^ T cells, priming cytotoxic effector CD8^+^ T cells. In addition, cDC1s within the tumor microenvironment produce the chemokines CXCL9/10 that can recruit CD8^+^ effector T cells into tumor tissue and can locally present tumor antigens to restimulate recruited T cells. Finally, the anti-tumor activity of T cells and NK cells within the tumor might further be boosted by cytokines made by cDC1, for example, interleukin-12 (IL-12), that, in turn, is amplified by T- and NK cell–derived cytokines such as interferon-γ (IFN-γ).

Besides CCL4, other chemokines can attract cDC1s into tumors. We recently investigated the accumulation of cDC1s in mouse transplantable tumors that are susceptible to cDC1-dependent immune control due to genetic ablation of the enzymes cyclooxygenase (COX)1 and COX2 in cancer cells 27, 31. In these COX-deficient tumors, the immune-suppressive prostanoid prostaglandin E_2_ (PGE_2_) is not produced, and cDC1s are recruited into the TME by the chemokines CCL5 and XCL1. Interestingly, those chemokines are not produced by the cancer cells but by natural killer (NK) cells that infiltrate tumors shortly after implantation [27]. NK cell–derived chemokines not only contribute to intratumoral cDC1 accumulation but also further regulate the local positioning of cDC1s within tumor tissue, allowing penetration of cDC1s into the TME and formation of NK cell/cDC1 clusters [27], an observation that was confirmed in an independent study [28]. Notably, in human cancer biopsies, the transcript levels of CCL5, XCL1, and its paralog XCL2 are associated with a cDC1-specific gene signature, suggesting a similar role for these chemokines in attracting cDC1s into human tumors [27]. The production of cDC1-recruiting chemokines by tumor NK cells therefore seems to be an important pathway regulating cDC1 accumulation within the TME (Figure 1).

Other cells such as CD8^+^ T cells and innate lymphocytes [e.g., γδ T cells, innate lymphoid cells (ILC1s)] are in principle able to produce both CCL5 and XCL1 and might contribute to cDC1 recruitment under certain circumstances or in other tumor contexts. In contrast to CCL5, which acts on the chemokine receptor CCR5 and can promote migration of tumor-promoting immune cells such as macrophages and T_reg_ cells 32, 33, XCL1 acts as a ligand for XCR1 and acts on XCR1^+^ cDC1s but not on other cells [7]. Local induction of XCL1 production in tumors, for example, by stimulation of intratumoral NK cells or targeted delivery of XCR1 ligands into tumors might therefore be an attractive strategy to attract cDC1s into the TME.

In addition to chemokine-mediated cDC1 recruitment and retention, cDC1 abundance within tumors is likely further regulated by the local availability of DC growth factors such as fms-like tyrosine kinase 3 ligand (FLT3L). Notably, NK cells were recently described as a key source of intratumoral FLT3L, which sustained the viability of cDC1s in the TME [28]. These data indicate that NK cells play a dual role in both cDC1 recruitment and positioning and in maintaining cDC1 longevity and functional competence 27, 28 (Figure 1). Notably, FLT3L can also act on cDC precursors to favor cDC1 differentiation [34]. Consistent with that notion, treatment of mice with FLT3L leads to accumulation in tumors of IRF8^+^CD11c^+^MHCII^+^CD103^−^cells that resemble precursors of cDC1 [11]. Intratumoral production of FLT3L by NK cells could therefore also facilitate local expansion of such precursors [28] (Figure 1). However, it is unlikely that all cDC1s in tumors originate from local expansion of recruited precursors. Indeed, fully differentiated cDC1 recruited from blood or surrounding tissue might be the predominant origin of intratumoral cDC1s that accumulate in response to XCL1 and CCL5 produced by tumor NK cells because cDC1 precursors express only low levels of transcripts for CCR5 and XCR1 [35] (Figure 1). Alternatively, the chemokines might be involved in prolonging intratumoral retention of cDC1s that differentiated locally from pre-cDCs, resulting in the observed net increase in cDC1 numbers.

## Priming of T Cells after Antigen Delivery to Tumor-Draining Lymph Nodes

*De novo* generation of cytotoxic effector CD8^+^ T cells specific for tumor antigens depends on cDCs cross-presenting tumor peptides on MHC class I molecules to naive antigen-specific T cells. Such T cell priming is thought to predominantly occur in tumor-draining lymph nodes, although some naive T cells might also be primed within the TME [36]. While tumor antigens can reach lymph nodes by themselves in certain experimental setups such as the injection of a high number of apoptotic tumor cells [37] or, naturally, during metastasis, priming of T cells in tumor-draining lymph nodes from progressively growing non-invasive tumors requires the delivery of tumor antigens to tumor-draining lymph nodes by migratory cDCs. Experiments with fluorescently labeled tumor cells demonstrated that although fluorescent material is efficiently taken up by different antigen-presenting cell populations within the TME, in tumor-draining lymph nodes fluorescence is predominantly detected in migratory CD103^+^ cDC1s 11, 38. It therefore seems that although tumor cDC2s also migrate to tumor-draining lymph nodes, only cDC1s are able to deliver intact tumor antigens to tumor-draining lymph nodes, a process that depends on the chemokine receptor CCR7 11, 38.

Within tumor-draining lymph nodes, CD103^+^ cDC1s transfer a fraction of fluorescently labeled material to other antigen-presenting cell populations, including resident CD8α^+^ cDC1s [38] (Box 2). Despite this transfer, when isolated from tumor-draining lymph nodes and tested *ex vivo*, only migratory CD103^+^ cDC1s, but not lymph node–resident CD8α^+^ cDC1s, have the capacity to stimulate naive CD8^+^ T cells against tumor-associated model antigens 11, 38. From these studies, it seems that priming of naive CD8^+^ T cells in tumor-draining lymph nodes relies on CD103^+^ cDC1s, but not other cDCs. Consistent with this view, the lack of CD8^+^ T cell priming towards tumor antigens observed in BATF3-deficient mice appears to be due to the absence of migratory CD103^+^ cDC1s rather than loss of lymph-node resident CD8α^+^ cDC1s 12, 39. The unique ability of migratory cDC1s to cross-present tumor antigens to CD8^+^ T cells might be related to reduced antigen degradation in phagocytic compartments [40], as well as the ability of cDC1s to use DNGR-1 to shuttle material from dead cells into specialized endocytic compartments [41]. The latter can fuse with endoplasmic reticulum–derived vesicles that contain the MHC class I loading machinery [42], a process that can be mediated by the SEC22b SNARE [43]. The role of DNGR-1 and SEC22b in anti-tumor immunity is under investigation 44, 45.

Box 2 **cDC1 Subsets in Lymph Nodes**

Two populations of cDC1s can be found in lymph nodes. Lymphoid-resident CD8α^+^ cDC1s develop locally from blood-borne precursors, while migratory CD103^+^ cDC1s enter via afferent lymphatics after migration from peripheral tissues or tumors. While these two cDC1 populations share similar gene expression signatures, and express cDC1-specific genes such as XCR1 and CLEC9A, migratory cDC1s in lymph nodes can be identified by higher expression of MHC class II. However, high levels of MHC class II expression can also be induced on CD8α^+^ cDC1s upon activation. In mice, only the selective expression of CD8α and CD103 allows for reliable discrimination of the two cDC1 subsets in lymph nodes.

Alt-text: Box 2

## A Local Role for DCs within the TME

Investigating a role for cDC1s in tumors is complicated by the fact that the lack of cDC1s in *Batf3*^–/–^ mice or their inability to migrate to lymph nodes in *Ccr7*^–/–^ mice abrogates priming of tumor-specific CD8^+^ T cells 11, 12. Nevertheless, several recent studies suggest that cDC1 plays an important role in regulating anticancer immune responses locally within tumor tissue (Figure 1).

Within tumors, CD103^+^ cDC1 appears to be the main source of the chemokines CXCL9 and CXCL10, key for the recruitment of CXCR3^+^ effector T cells [46], and for facilitating T cell–mediated control of melanoma growth [14]. The cDC1-dependent guidance of effector T cells into tumors might be the reason why cDC1s are required for the responsiveness of cancer to adoptive T cell therapy [10]. In addition, production of CXCL9/10 might be important for positioning tumor-infiltrating T cells in cDC1-rich areas and facilitating local T cell restimulation, similar to the positioning of memory CD8^+^ T cells by CXCL9 in lymph nodes upon viral infection [47]. Of note, CXCL9 and CXCL10 expression in myeloid cells is not constitutive but requires exposure to type I interferon (IFN) or IFN-γ, suggesting a positive feedback loop whereby an influx of IFN-γ–producing CD8^+^ T cells into the TME could amplify cDC1-dependent recruitment of additional T cells.

The ability to acquire tumor material is not restricted to cDC1 but can be observed by several myeloid cell populations, including tumor-associated macrophages 10, 11, 48. However, when analyzed *ex vivo*, tumor cDC1s are superior to other myeloid cells in stimulating T cell activation and proliferation, suggesting that they process ingested antigens more efficiently [10]. Furthermore, cDC1s produce high amounts of the cytokine interleukin (IL)-12 10, 25, which likely helps sustain the cytotoxic function of CD8^+^ effector T cells within the TME (Figure 1).

It seems likely that a stimulatory role for cDC1 in tumors is not restricted to T cells. Production of chemokines such as CXCL9 and CXCL10 might result in the recruitment of other CXCR3^+^ cells such as NK cells or ILC1 into tumors [49], which, by producing XCL1/2 and FLT3L facilitate close cell appositioning and promote reciprocal interactions. Local production of IL-12 by cDC1 supports NK cell production of IFN-γ and control of lung metastasis [50] and, in turn, IFN-γ potentiates IL-12 production by cDC1s [51]. One could envision that this two-way dialog extends to a three-partner conversation and that the local interaction between NK cells, cDC1s, and, eventually, T cells provides a localized cytokine milieu that favors anti-tumor immunity (Figure 1). Such interactions, if validated, have important implications for the design of NK cell and T cell–based immunotherapies.

The dynamics of cDC1 trafficking within tumors remains enigmatic, and it is currently not known whether all cDC1s that enter tumors will eventually leave and migrate to tumor-draining lymph nodes [38]. It might well be that some cDC1s establish residence in tumors and orchestrate local anti-tumor immunity by presenting tumor antigens to incoming T cells and stimulating NK and other innate immune cells, as stated above. It will be important to determine the half-life of cDC1s within tumors, especially of those cells that have acquired and processed tumor antigens for presentation to T cells, and the kinetics of cDC1 production of proinflammatory and immunomodulatory cytokines.

## Impairment of cDC Function

During cancer development, tumor cells interact with surrounding cells to establish a local immunosuppressive milieu, allowing cancers to evade detection and destruction by the immune system [52]. Such an environment not only impairs tumor-specific T cells but also affects cDC1 biology, thereby also limiting anti-tumor immunity.

The exclusion of cDC1s from tumors is emerging as an important cancer immune evasion strategy. Low cDC1 abundance in tumors might result from reduced development of cDC1s, for example, through systemic suppression of DC development in the bone marrow [53] or by limiting the local production of FLT3L and other growth factors important for cDC1 differentiation, expansion, and survival 11, 28. In addition, the scarcity of tumor cDC1s might further ensue from suppression of chemokine-mediated recruitment of cDC1s into the TME. For example, we observed that PGE_2_ produced by tumor cells acts on both NK cells and cDC1s to suppress production of cDC1 chemoattractants by the former and responsiveness to the chemokines by the latter [27]. As already mentioned, loss of production of CCL4 in β-catenin^+^ tumors might be responsible for reduced cDC1 recruitment [13]. Similarly, the loss of the tumor suppressor *TRP53* results in reduced production of CCL3, CCL4, and CCL5 by cancer cells [54], chemokines that potentially mediate cDC1 recruitment into tumors. It remains to be seen whether a shift in the chemokine profile towards cDC1-attracting chemokines can generally be induced by interference with oncogenic signaling in cancer cells.

Inhibitory factors present within the TME not only regulate cDC1 access to tumor tissue but also directly act on cDC1s by limiting their stimulatory activity. For example, in breast cancer the production of IL-10 by tumor-associated macrophages suppresses the production of IL-12 by cDC1s [25]. Similarly, cDC1s in PGE_2_-producing BRAF^V600E^ melanoma display reduced IL-12 production and fail to express co-stimulatory molecules [31]. Furthermore, tumor-derived factors can induce the intracellular accumulation of oxidized lipids in cDC1s, resulting in impaired antigen cross-presentation to CD8^+^ T cells 55, 56. Other tumor-derived factors such as transforming growth factor β (TGF-β) might similarly affect the anti-tumor activity of cDC1s. *In vitro*, TGF-β can inhibit ability of myeloid cells to take up antigen and secrete cytokines and chemokines, and blockade of TGF-β signaling has been shown to improve the efficacy of DC cancer vaccines 57, 58. However, a major target of TGF-β is the tumor stromal compartment [59], and it has yet to be established how TGF-β affects the cDC1 subset in tumors and the relevance of such suppression for anti-tumor immunity *in vivo*.

The activity of cDC1s in tumors might be further limited by signals from immune inhibitory receptors expressed on cDC1s. Although little is known about the relevance of PD-1 expression on cDCs, human cDC1s circulating in the blood of hepatocellular carcinoma patients show increased expression of that inhibitory receptor, suggesting that they might be susceptible to signaling in response to PD-L1 expressed on cells within the TME [60]. In the murine MMTV-PyMT breast cancer model, cDC1s do not display elevated PD-1 expression but highly express the immune inhibitory receptor TIM-3 [61]. Antibody-mediated blockade of TIM-3 or its ligand, galectin 9, increases CXCL9 production in cDC1s and results in cDC1-dependent immune control. While these findings suggest that TIM-3 signaling on cDC1s in breast cancer inhibits their ability to recruit CXCR3^+^ T cells into the TME, anti–TIM-3 treatment does not lead to an increase in intratumoral T cells [61], indicating additional effects that remain to be investigated.

With respect to the metabolic demands of immune cells, the TME constitutes a challenging environment with limited availability of oxygen and nutrients and increased concentration of metabolic products such as lactate due to aerobic glycolysis in proliferating cancer cells [62]. During their activation, DCs undergo substantial metabolic reprogramming to meet demands for protein synthesis and secretion, characterized by an increase in glucose uptake and enhanced glycolysis [63]. Although cDC1 metabolism has yet to be extensively studied, competition for glucose with other cells in the TME could dampen the ability of cDC1 to produce chemokines and cytokines in response to their activation in tumors, similar to the metabolic restriction imposed on effector T cells in tumors [64]. In addition, lactate has been shown to inhibit the secretion of cytokines by monocyte-derived DCs [65]. It is therefore possible that the high levels of lactate produced by tumor cells could impact on the production of chemokines and cytokines by tumor cDC1s *in vivo* [66]. Future studies will be necessary to elucidate the regulation of cDC1 metabolism in tumors and the impact of such regulation on their ability to orchestrate anti-tumor immunity.

## Concluding Remarks

cDC1s are critically involved in the initiation of tumor-specific T cell responses in tumor-draining lymph nodes. However, recent evidence suggests a fundamental role for cDC1 in the regulation of cancer immunity and the immune cell composition within the tumor microenvironment, with important consequences for cancer immunotherapy. This local role involves the regulation of cytotoxic T cell recruitment and restimulation, but probably extends to other immune cell subsets within tumors, including NK cells. We still know very little about the biology of cDC1 in tumors, especially in human cancer patients. Future studies will help increase our knowledge of the multiple functions of DCs within the complex tumor microenvironment (see Outstanding Questions), from the acquisition of tumor antigens to local trafficking and communication with other immune cells. It will be important to further characterize the mechanisms by which different oncogenic signaling pathways, immunosuppressive factors secreted by tumor cells, and the special metabolic environment within tumors impact on the diverse aspects of cDC1 function. Strategies that aim to enhance the abundance and function of cDC1s in tumors may provide promising new ways to improve the responsiveness of cancer patients to immunotherapy.

Outstanding Questions By which mechanisms and pathways do cDC1s regulate anti-tumor immunity within the tumor microenvironment and during immunotherapies such as immune checkpoint blockade?

Which mechanisms and local factors regulate cDC1 function within the microenvironment of tumors?

To what degree can cDC1 abundance in tumors serve as a predictive biomarker for the outcome of cancer immunotherapies?

Which strategies can be used to increase cDC1 numbers in tumors to enhance anti-tumor immunity and responsiveness to checkpoint blockade?

Are therapies targeting NK cells a promising approach to promote intratumoral cDC1 recruitment and survival?
